# Metabolomics Analysis of Functional Activity Changes in Residual Tumour Cells After IOCS Treatment

**DOI:** 10.1111/jcmm.70452

**Published:** 2025-03-20

**Authors:** Lai‐wei You, Jinhuo Wang, Dan Yin, Bao‐ji Hu, Yong Cheng, Xue‐fei Wang, Hao Li, Jianrong Guo

**Affiliations:** ^1^ School of Clinical Medicine Ningxia Medical University Ningxia China; ^2^ Postgraduate Training Base in Shanghai Gongli Hospital Ningxia Medical University Shanghai China; ^3^ Department of Anesthesiology Gongli Hospital of Shanghai Pudong New Area Shanghai China; ^4^ Department of Anesthesiology Shanghai Pudong Hospital, Fudan University Pudong Medical Center Shanghai China

**Keywords:** hepatocellular carcinoma, intraoperative cell salvage, metabolomics analysis

## Abstract

Hepatocellular carcinoma (HCC) is a serious and often lethal cancer, particularly in patients with chronic liver disease. Currently, no specific treatment has been utilised to prevent HCC. The detailed mechanism of HCC is still elusive, and this study aims to identify and characterise the functional activity changes in residual tumour cells following intraoperative cell salvage (IOCS) treatment during HCC surgery. This research is a retrospective case–control study, involving the selection of 60 patients with HCC who underwent radical surgery; then blood and tumour tissue were collected for further testing. GC–MS assay, immunofluorescence, Western blot and qRT‐PCR techniques were employed. Our study found comparable demographic and baseline clinical characteristics between the experimental group (*n* = 30), which received IOCS treatment during surgery, and the control group (*n* = 30), which did not receive IOCS treatment, validating subsequent analyses. Metabolomic analysis revealed six key metabolites differing between groups, indicating improvement in liver tumours in the experimental group. TP53 expression was significantly upregulated, potentially mediating therapeutic effects. The intervention reduced HCC cell migration and apoptosis, decreased E2F1 and MDM2 protein and mRNA levels, and increased TP53 and CTNNB1 levels. These findings support the potential clinical application of the intervention in improving treatment outcomes for HCC patients, warranting further investigation to elucidate the underlying mechanisms and optimise therapeutic strategies.

## 1 Introduction

Hepatocellular carcinoma (HCC) represents the most common type of liver cancer, originating from hepatocytes, which are the liver's main cell type. In the early phases, it might be symptomless or exhibit vague symptoms such as tiredness, weight loss and abdominal discomfort [1, 2]. In advanced stages, symptoms like jaundice, ascites and hepatic encephalopathy may appear [3]. Diagnosis is made through imaging methods, blood tests and biopsy [4, 5]. HCC is the sixth most prevalent cancer globally and stands as the third primary reason for deaths associated with cancer. Its incidence is higher in regions like East Asia and sub‐Saharan Africa, largely due to widespread hepatitis B and C infections [2]. The high mortality rate of HCC is attributed to its late detection and aggressive progression, often invading blood vessels and causing intrahepatic spread and distant metastasis [6]. Advanced HCC can result in liver failure, complicating treatment and increasing death rates. Surgical removal of part of the liver can be curative in early‐stage HCC, but only a small fraction of patients qualifies for this [1]. Many HCC cases are identified at an advanced stage, where curative interventions are no longer feasible [3]. Systemic therapies like sorafenib offer limited survival benefits and can have significant side effects, with high recurrence rates following treatment, especially after resection and ablation. HCC poses a major global health issue due to its aggressive nature, high mortality, and the complexities involved in its treatment. Enhancing early detection, improving treatment efficacy, and ensuring better access to care are essential in improving outcomes for HCC patients.

Intraoperative cell salvage (IOCS) is a surgical procedure that entails the collection, purification and reinfusion of a patient's own blood that is lost during surgery [7]. This approach aims to decrease the reliance on allogeneic blood transfusions, which pose risks like immune reactions and infections [8]. Surgeries on the liver, such as those for HCC, can result in substantial blood loss [5]. IOCS addresses this issue by reducing the need for donor blood and the associated transfusion risks. However, a major concern with using IOCS in cancer surgeries, including HCC, is the potential reinfusion of tumour cells collected along with the blood [9, 10]. Reinfusing blood that contains residual tumour cells could theoretically lead to cancer recurrence or metastasis. Modern IOCS systems incorporate filtration and washing steps to remove tumour cells and other impurities from the salvaged blood before reinfusion.

Residual tumour cells that survive surgery and filtration processes may undergo functional changes, allowing them to adapt and thrive in new environments [11]. These changes can potentially increase the metastatic potential of the residual tumour cells, making them more aggressive and capable of forming new tumour sites [12]. Some residual tumour cells may undergo epithelial‐mesenchymal transition (EMT), enhancing their migratory and invasive abilities [13]. Others may acquire stem cell‐like properties, contributing to tumour recurrence and resistance to conventional treatments. While IOCS is beneficial for managing blood loss during HCC surgery, it carries the risk of reinfusing residual tumour cells [14]. Functional changes in these cells can increase their survival and metastatic potential, necessitating comprehensive strategies to mitigate these risks and vigilant postoperative monitoring [15]. Advances in filtration technology, adjuvant therapies and personalised medicine are essential to optimising the advantages of IOCS while minimising the associated risks of residual tumour cells [11].

The primary objective of this study is to conduct a comprehensive metabolomics analysis to identify and characterise the functional activity changes in residual tumour cells following IOCS treatment during HCC surgery.

## 2 Materials and Methods

### 2.1 Patients

This study was designed as a retrospective case–control study. A total of 60 patients diagnosed with HCC who underwent radical surgery at our hospital between January 2022 and December 2023 were included. Informed consent was obtained from all patients, and the study was approved by the hospital's ethics committee. Patients were randomly assigned to one of two groups: experimental group (*n* = 30): Received IOCS treatment during surgery. Control group (*n* = 30): did not receive IOCS treatment. Sample collection and grouping blood and liver tumour tissue samples were collected during the surgical procedure, specifically at the time of tumour resection. The details of sample collection are as follows: blood in the experimental group was collected directly from the surgical site during tumour resection and immediately transferred to a storage tank using the IOCS device. This blood underwent centrifugation, separation, washing and filtration through a leukocyte filter for further processing. Blood in the control group was also collected from the surgical site but was not processed using the IOCS device. Instead, blood samples were stored immediately for analysis without any filtration, washing, or processing. In addition to blood samples, tumour tissue excised from the liver during surgery was also collected for analysis from both groups. Timing of sample collection all samples were collected intraoperatively, specifically during the surgery at the time of tumour resection. The exact time point of collection was directly tied to the surgical procedure and the removal of the tumour. The timing of blood collection was recorded in detail, as this is crucial to understanding the perioperative effects of the IOCS treatment. In the experimental group, blood was processed immediately following collection from the surgical site, using the IOCS device to undergo centrifugation, separation and filtration. In the control group, blood was collected from the surgical site and stored directly, without any additional processing, filtration, or washing. Since both blood and tumour tissue samples were obtained simultaneously during the surgery, the study primarily focuses on capturing the immediate perioperative effects of IOCS treatment on tumour metabolism and cell behaviour, rather than on longer‐term effects that might occur postsurgery. The key point of sample collection in our study is the immediacy of the perioperative timing. All biological samples—both blood and liver tumour tissue—were collected during the surgical procedure in real time, ensuring that any observed effects in cell behaviour and tumour metabolism are reflective of the immediate intraoperative changes, rather than changes that might occur hours or days after surgery. This timing is essential to understanding the direct impact of IOCS on tumour biology during surgery. All participants were fully informed about the study and provided written consent, as was approved by the Ethics Committee of Gongli Hospital of Shanghai Pudong New Area, Shanghai, China.

Inclusion criteria: (1) patients aged 20 years or older; (2) confirmed preoperative diagnosis and postoperative histopathological verification of liver cancer; (3) undergoing radical liver cancer resection; (4) significant intraoperative blood loss necessitating blood transfusion; (5) no preoperative anaemia; (6) liver function classified as Child–Pugh grade A or B.

Exclusion criteria: (1) presence of concurrent heart or kidney failure; (2) undergoing minor resection of the liver surface with minimal blood loss; (3) concurrent coagulation disorders or haematological diseases; (4) incomplete clinical data.

### 2.2 GC‐MS Assay

Blood samples were collected from the HCC patients, and serum was separated by centrifugation at 3000 rpm for 10 min at 4°C. The supernatant (serum) was carefully aspirated and stored at −80°C until further analysis. Serum constituents were examined using a GC–MS system (7890A/5975C, Agilent Technologies, USA). Samples were analysed using a gas chromatography–mass spectrometry (GC–MS) system. High‐purity helium (purity > 99.999%) was utilised as the carrier gas, with a constant flow rate of 1 mL/min. The GC oven temperature programme was as follows: Initial temperature: 70°C, held for 2 min; ramped to 160°C at a rate of 6°C/min; increased to 240°C at a rate of 10°C/min; finally raised to 300°C at a rate of 20°C/min and held for 6 min. Sample injection was performed in split mode with a split ratio of 2:1. The injection volume was 1 μL, and a solvent delay of 6 min was applied. The inlet temperature was set at 250°C. Mass spectrometry conditions were as follows: Transfer line temperature: 260°C; electron impact (EI) ion source temperature: 230°C; EI energy: 70 eV; data acquisition: Full‐scan mode; mass‐to‐charge ratio (m/z) range: 50–600.

### 2.3 HE Staining

Liver tumour tissue samples were fixed in 10% neutral‐buffered formaldehyde (pH 7.4) for 24 h at room temperature to preserve tissue morphology and prevent degradation. This concentration of formaldehyde is commonly used for tissue fixation and ensures effective preservation of cellular structures. After fixation, the tissue samples were processed through a series of dehydration steps using increasing concentrations of ethanol, followed by paraffin embedding. The samples were then cut into thin sections, each 5 μm thick, using a microtome. The tissue sections were mounted onto glass slides and subjected to staining with haematoxylin and eosin (HE) following standard protocols: haematoxylin staining was carried out for 5 min to stain the nuclei blue. The slides were then washed in running tap water for 2 min to remove excess haematoxylin. Afterward, the sections were stained with eosin for 2 min to stain the cytoplasm and extracellular matrix structures a pink colour. Following the staining process, the slides were cleared in xylene and mounted with a coverslip using a mounting medium. A pathology expert then examined the stained tissue sections under a light microscope to assess the histological findings, including cellular morphology, tumour differentiation and tissue architecture.

### 2.4 Immunofluorescence Assay

The immunofluorescence assay was performed as follows: stabilisation and infiltration: liver tumour tissue samples were first subjected to stabilisation and infiltration techniques to prepare the tissue for antibody binding. This step ensures proper tissue fixation and permeabilisation. Primary antibody incubation: the tissue sections were then immersed in a bath containing the primary antibodies. The primary antibodies were diluted in an appropriate blocking buffer and incubated with the samples for 1 h at room temperature. Washing: after primary antibody incubation, the samples were promptly rinsed with phosphate‐buffered saline (PBS) to remove any unbound antibodies. The tissue was washed three times, each for 5 min. Secondary antibody incubation: next, the samples were incubated with fluorescently labelled secondary antibodies (appropriate for the species of the primary antibody). This incubation was performed for 45 min at room temperature in the dark to prevent photobleaching. Washing: after the secondary antibody incubation, the samples were washed again with PBS three times for 5 min each to remove excess unbound secondary antibodies. Microscopy: finally, the tissue sections were mounted with a mounting medium containing DAPI (4′,6‐diamidino‐2‐phenylindole) for nuclear staining and then examined under a fluorescence microscope to visualise the immunofluorescent signals and analyse the cellular localisation of the proteins of interest.

### 2.5 Transwell Assay

To assess the invasive properties of HCC cells, liver tumour cells were first suspended in serum‐free medium. A 200 μL aliquot of the cell suspension was then added to a Transwell insert precoated with a thin layer of Corning Matrigel Matrix (Catalogue Number: 354234). The bottom chamber was filled with 600 μL of nutrient‐enriched medium containing 5% foetal bovine serum (FBS) (Catalogue Number: 16000044; Thermo Fisher Scientific), which provided essential nutrients and growth factors necessary for cell migration. After 24 h of incubation at 37°C in a humidified 5% CO₂ incubator, the cells that had migrated through the matrix and the membrane were fixed with 4% paraformaldehyde for 15 min. Nonmigratory cells were removed from the upper surface of the membrane using a cotton swab. The invasive cells were then stained with crystal violet (0.1% in methanol) for 20 min and washed with PBS. The invasive cells that had traversed the matrix were captured and examined using a Leica DMi8 Optical Microscope (Catalogue Number: 10422308) with appropriate magnification. The number of invasive cells was quantified by counting the cells in five random fields per membrane. For data analysis, flow cytometry was performed using the BD FACSCanto II Flow Cytometer (BD Biosciences, Catalogue Number: 643798) to evaluate cellular characteristics associated with migration. FlowJo v10 (FlowJo LLC) software was used to analyse the flow cytometry data.

### 2.6 Flow Cytometry Assay

The isolated tumour cells were subjected to flow cytometry analysis following the manufacturer's instructions. After collection, the tissues were stained with Annexin V‐FITC and Propidium Iodide (PI) in a dark environment. Subsequently, apoptosis levels in all groups were quantified using a flow cytometer.

### 2.7 Western Blotting

Protein extracts from liver tumour tissues were resolved by SDS‐PAGE (10% gel), and the separated proteins were transferred to PVDF membranes (Millipore, Catalogue Number: IPVH00010). After transfer, the membranes were rinsed with TBST buffer (Tris‐buffered saline with 0.1% Tween‐20, pH 7.4) to remove any nonspecifically bound proteins. Blocking: The membranes were blocked with 5% nonfat milk in TBST for 1 h at room temperature to prevent non‐specific binding. The membranes were incubated overnight at 4°C with the following primary antibodies: Primary antibody for target proteins: E2F1, MDM2, TP53 and CTNNB1 (Bioworld Technology Inc., China) diluted at 1:1000 in blocking buffer. Primary antibody for actin: Anti‐β‐actin (Bioworld Technology Inc., China) diluted at 1:5000 in blocking buffer. After primary antibody incubation, the membranes were washed three times with TBST for 5 min each to remove any unbound primary antibodies. The membranes were then incubated with the appropriate horseradish peroxidase (HRP)‐conjugated secondary antibodies: Secondary antibody for target proteins: HRP‐conjugated antirabbit IgG (Bioworld Technology Inc., China), diluted at 1:2000 in blocking buffer. Secondary antibody for actin: HRP‐conjugated antimouse IgG (Bioworld Technology Inc., China), diluted at 1:5000 in blocking buffer. The secondary antibody incubation was carried out for 2 h at room temperature. The membranes were washed again three times with TBST for 5 min each to remove any residual unbound secondary antibodies. Protein Detection: Protein bands were visualised using ECL chemiluminescence reagent (Thermo Fisher Scientific, Catalogue Number: 34095) according to the manufacturer's instructions. The chemiluminescent signal was detected using a chemiluminescence imager (e.g., Bio‐Rad ChemiDoc MP Imaging System). The bands were analysed using Image Lab Software (Bio‐Rad), and protein expression levels were quantified relative to the β‐actin loading control.

### 2.8 qRT‐PCR

Total RNA was isolated from liver tumour samples using the TRIzol Reagent (Beyotime, Shanghai) following the manufacturer's instructions. The mRNA was then converted into cDNA utilising the mRNA Reverse‐Transcription Kit (Beyotime, Shanghai). To quantify mRNA expression, quantitative PCR was conducted with the SYBR Green PCR Mix (Vazyme Biotech, Shanghai) on a Real‐Time PCR System. The relative expression levels were determined using the 2^−ΔΔ*Ct*^ method, with normalisation to GAPDH. This experiment was repeated three times. The primers used were as follows: for E2F1, Forward: 5′‐GCAGAGCAGATGGTTATGG‐3′ and Reverse: 5′‐ACTATGGTGGCAGAGTCAG‐3′; for MDM2, Forward: 5′‐TCACGCCTGTAATCTCAAC ‐3′ and Reverse: 5′‐AGTCTCCATCTGTCTCCTAAG‐3′; for TP53, Forward: 5′‐GTGAGGGATGTTTGGGAGATG‐3′ and Reverse: 5′‐CCTGGTTAGTACGGTGAAGTG‐3′; for CTNNB1, Forward: 5′‐TACCTCCCAAGTCCTGTATG‐3′ and Reverse: 5′‐TGATGGTTCAGCCAAACG‐3′; and for GAPDH, Forward: 5′‐CACCCACTCCTCCACCTTTG‐3′ and Reverse: 5′‐CCACCACCCTGTTGCTGTAG‐3′.

### 2.9 Statistical Analysis

Statistical analyses were performed using Prism 8 software. The data were presented as the mean ± SD, with each experiment being repeated a minimum of three times. To determine differences between two groups, a t‐test was utilised. For comparisons among three or more groups, a one‐way ANOVA was employed. A *p* value less than 0.05 was deemed statistically significant. Metabolomics Data Pre‐processing and Validation: Data Normality: The underlying metabolomics data were assessed for normality using the Shapiro–Wilk test. This test was applied to each set of data to confirm whether the data followed a normal distribution. The results of this test are provided in the Supporting Information for reference. Preprocessing steps: prior to statistical analysis, the metabolomics data underwent log transformation to stabilise the variance and normalise the data distribution. This transformation was applied to the raw intensity values of metabolites to correct for skewness and to make the data more suitable for subsequent statistical analysis. Additional pre‐processing steps, including data scaling (e.g., autoscaling or Pareto scaling), were applied as needed, depending on the specific requirements of the downstream analysis. Outlier Detection: Outliers were detected using Grubbs' test or the IQR method, and any identified outliers were excluded from the analysis to ensure the integrity of the data.

### 2.10 Participants General Data

The demographic and baseline clinical characteristics of the HCC patients (shown in Table 1). Statistical analyses were conducted to compare the two groups, ensuring comparability for the study outcomes. Comparative analysis revealed no statistically significant differences in terms of gender distribution (*χ* ^2^ = 0.600, *p* = 0.439), age (*t* = 0.182, *p* = 0.856), body mass index (BMI) (*t* = 0.985, *p* = 0.329), Child–Pugh classification (*χ* ^2^ = 0.625, *p* = 0.429), alkaline phosphatase (ALP) levels (*t* = 0.476, *p* = 0.636), gamma‐glutamyl transferase (GGT) levels (*t* = 0.442, *p* = 0.660), alanine aminotransferase (ALT) levels (*t* = 0.222, *p* = 0.825), aspartate aminotransferase (AST) levels (*t* = 0.125, *p* = 0.901), total bilirubin levels (*t* = 0.175, *p* = 0.862) and tumour, node, metastasis (TNM) stage distribution (*χ* ^2^ = 0.362, *p* = 0.834). In the experimental group (IOCS‐treated), the average percentage of residual tumour cells in the liver tissue was significantly lower compared to the control group. Specifically, 10.5% of the tumour area contained residual tumour cells in the experimental group, whereas the control group had an average of 18.3% residual tumour cells. This reduction in residual tumour cells in the experimental group was statistically significant (*p* < 0.05), suggesting that IOCS treatment may help reduce the number of viable tumour cells remaining in the liver after surgery. These results indicate that the two groups were comparable in terms of demographic and baseline clinical characteristics, which supports the validity of subsequent analyses on treatment outcomes.

**TABLE 1:** Demographic, participants general data.

Variable	Experimental group (*N* = 30)	Control group (*N* = 30)	*χ* ^2^/*t*	*p*
Gender, male (%)	18 (60%)	15 (50%)	0.600	0.439
Age (years)	63.4 (46.3 – 72.6)	62.7 (45.4 – 73.2)	0.182	0.856
Body mass index (kg/m^2^)	27.3 (23.6 – 29.1)	26.6 (23.1 – 30.8)	0.985	0.329
Child‐Pugh classification			0.625	0.429
A	21 (70%)	18 (60%)		
B	9 (30%)	12 (40%)		
ALP (U/L)	150.6 (121.4 – 186.2)	148.2 (119.6 – 183.8)	0.476	0.636
GGT (U/L)	81.3 (62.4 – 97.8)	79.2 (61.8 – 101.3)	0.442	0.660
ALT (U/L)	60.5 (40.2 – 81.8)	61.8 (41.4 – 81.6)	0.222	0.825
AST (U/L)	56.2 (40.2 – 76.1)	55.6 (40.6 – 77.3)	0.125	0.901
Total bilirubin (mg/dL)	3.2 (1.5 – 4.7)	3.4 (1.4 – 4.5)	0.175	0.862
TNM stage			0.362	0.834
I	16	14		
II	13	15		
III	1	1		
IV	0	0		

Abbreviations: ALP, alkaline phosphatase; ALT, alanine aminotransferase; AST, aspartate aminotransferase; GGT, gamma‐glutamyl transferase; TNM, tumour, node, metastasis.

### 2.11 Identification of Differential Metabolites

**FIGURE 1:**
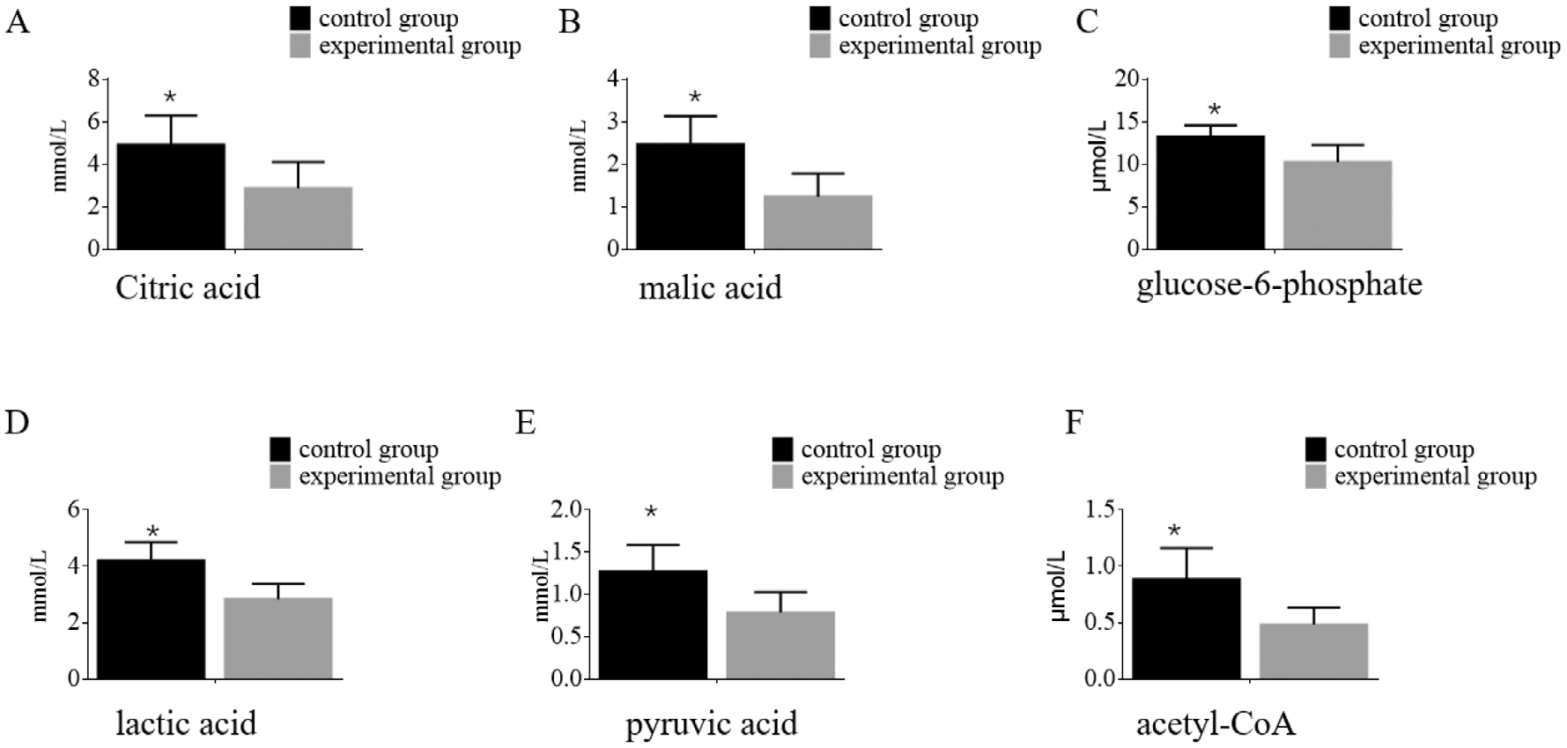
Identification of differential metabolites in blood samples between two patient groups using GC–MS. *p indicates that the difference is statistically significant (*p* < 0.05) when compared to the control group.

Metabolomic profiling of blood samples from the experimental and control groups was conducted using GC–MS technology. The analysis identified six key differential metabolites between the groups, including citric acid, malic acid, glucose‐6‐phosphate, lactic acid, pyruvic acid and acetyl‐CoA, which were found in significantly higher levels in the control group when compared to the experimental group, with all observed differences being statistically significant (*p* < 0.05). These findings are summarised in Figure 1. These results indicate a distinct metabolic profile in the control group characterised by elevated levels of specific metabolites, suggesting potential metabolic alterations associated with the conditions being studied. The significant differences in metabolite levels underscore the importance of these biomarkers in differentiating between the experimental and control groups.

### 2.12 Histopathological Examination Results

**FIGURE 2:**
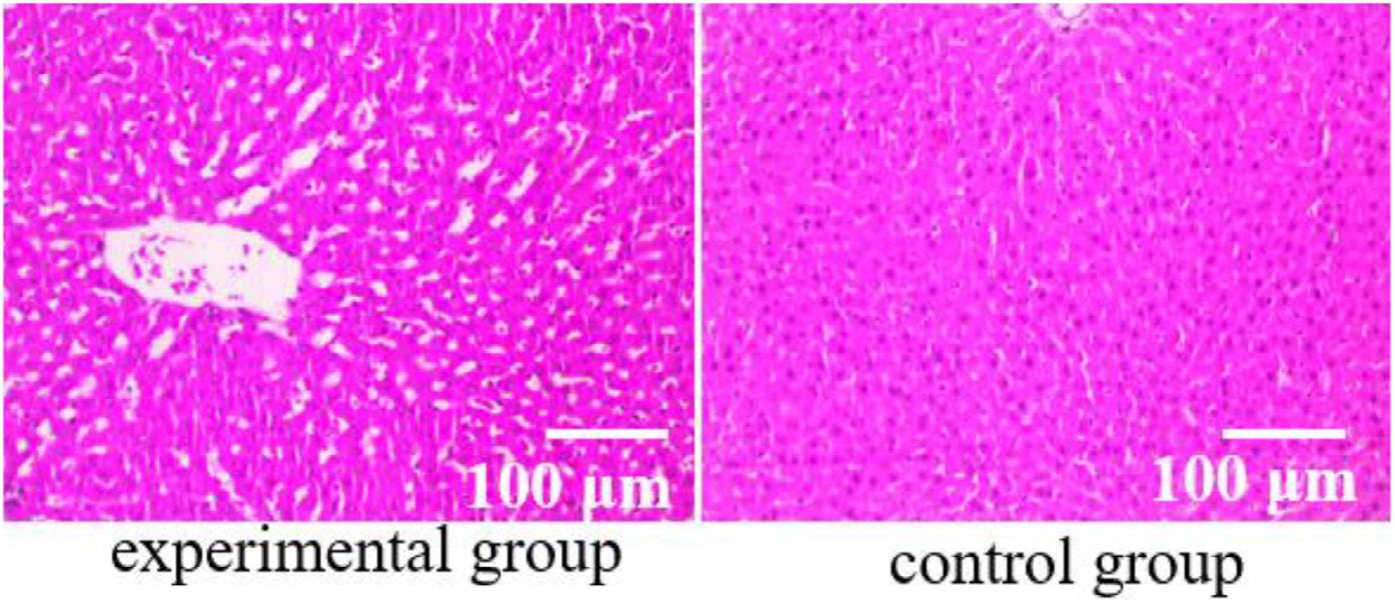
Histopathological examination of liver tumour tissues in HCC patients was conducted using HE staining.

Histopathological examination of liver tumour tissues was conducted using HE staining to assess the pathological condition of hepatocytes in patients (shown in Figure 2). The analysis revealed that the pathological test results for participants in the experimental group showed significantly better outcomes compared to the control group. Quantification of pathological features such as necrosis, inflammatory infiltration and fibrosis was performed using histopathological scoring systems. Liver tissues in the experimental group exhibited significantly fewer signs of necrosis (*p* < 0.05), reduced inflammatory infiltration (*p* < 0.01), and lower degrees of fibrosis (*p* < 0.01) when compared to the control group, as quantified by ImageJ analysis and blinded assessment by two independent pathologists. Additionally, the architecture of hepatocytes in the experimental group appeared more preserved, with significantly fewer abnormalities observed in tissue structure and cell morphology (*p* < 0.05), as assessed through quantitative analysis of histological sections. These findings highlight a marked improvement in the pathological condition of liver tumour tissues in the experimental group, indicating a beneficial effect of the intervention on liver pathology compared to the control group.

### 2.13 The Effect of TP53 in HCC Patients

**FIGURE 3:**
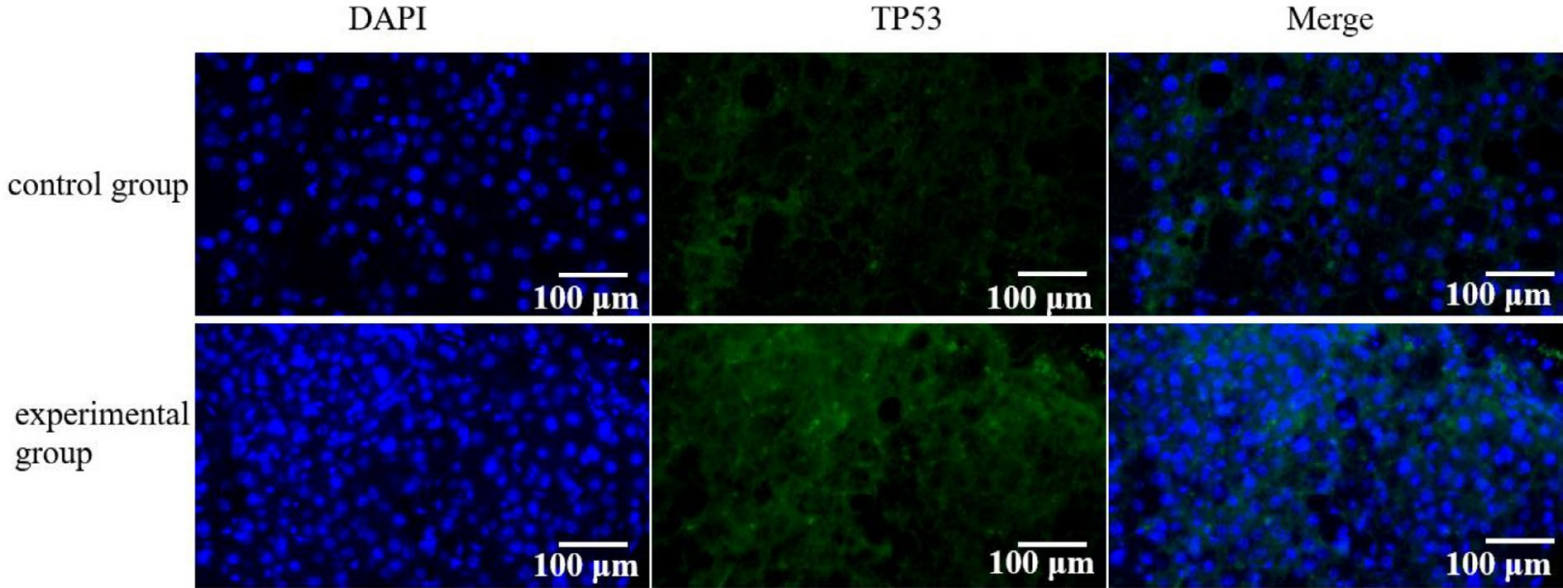
The effect of TP53 in HCC patients was detected using immunofluorescence assay.

To assess TP53 expression in liver tumour tissues from HCC patients, an immunofluorescence assay was employed. A total of *n* = 10 samples from each group (experimental and control) were analysed. The findings revealed a marked increase in TP53‐positive cells in the liver tumour tissues of the experimental group compared to the control group (*p* < 0.05). These findings are presented in Figure 3. The figure shows representative immunofluorescence images of TP53 expression in liver tumour tissues from both experimental and control groups. The experimental group displays a higher density of TP53‐positive cells, indicated by the fluorescent markers, compared to the control group. These findings indicate that the intervention in the experimental group is associated with a significant upregulation of TP53 expression in liver tumour tissues, suggesting a potential mechanism of action through TP53‐mediated pathways in HCC patients.

### 2.14 Transwell Assay Results

**FIGURE 4:**
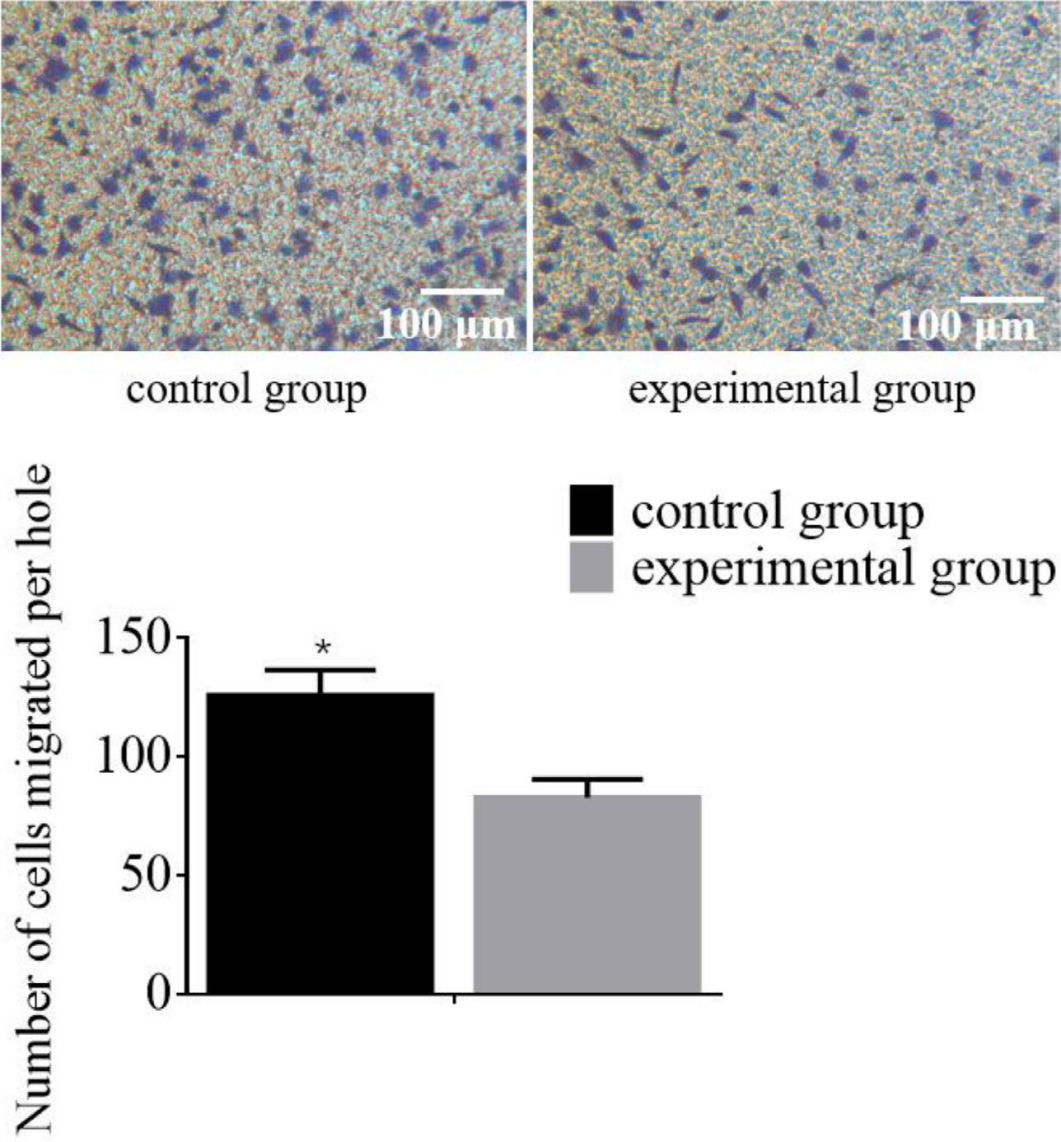
The migration ability of HCC cells was detected using the Transwell assay. *p indicates that the difference is statistically significant (*p* < 0.05) when compared with the control group.

The migratory capacity of HCC cells was evaluated via a Transwell assay. The findings demonstrated that the experimental group showed a marked reduction in HCC cell migration compared to the control group, with a *p* value below 0.05. These findings are illustrated in Figure 4. The figure shows representative images from the Transwell assay, and this underscores the number of migrated HCC cells in both experimental and control groups, with the experimental group displaying a significantly lower count of migrated cells compared to the control group. These results demonstrate that the experimental intervention effectively inhibits the migratory capability of HCC cells, suggesting a potential therapeutic benefit in reducing the invasiveness of HCC.

### 2.15 Comparison of Apoptosis Levels in HCC Cells

**FIGURE 5:**
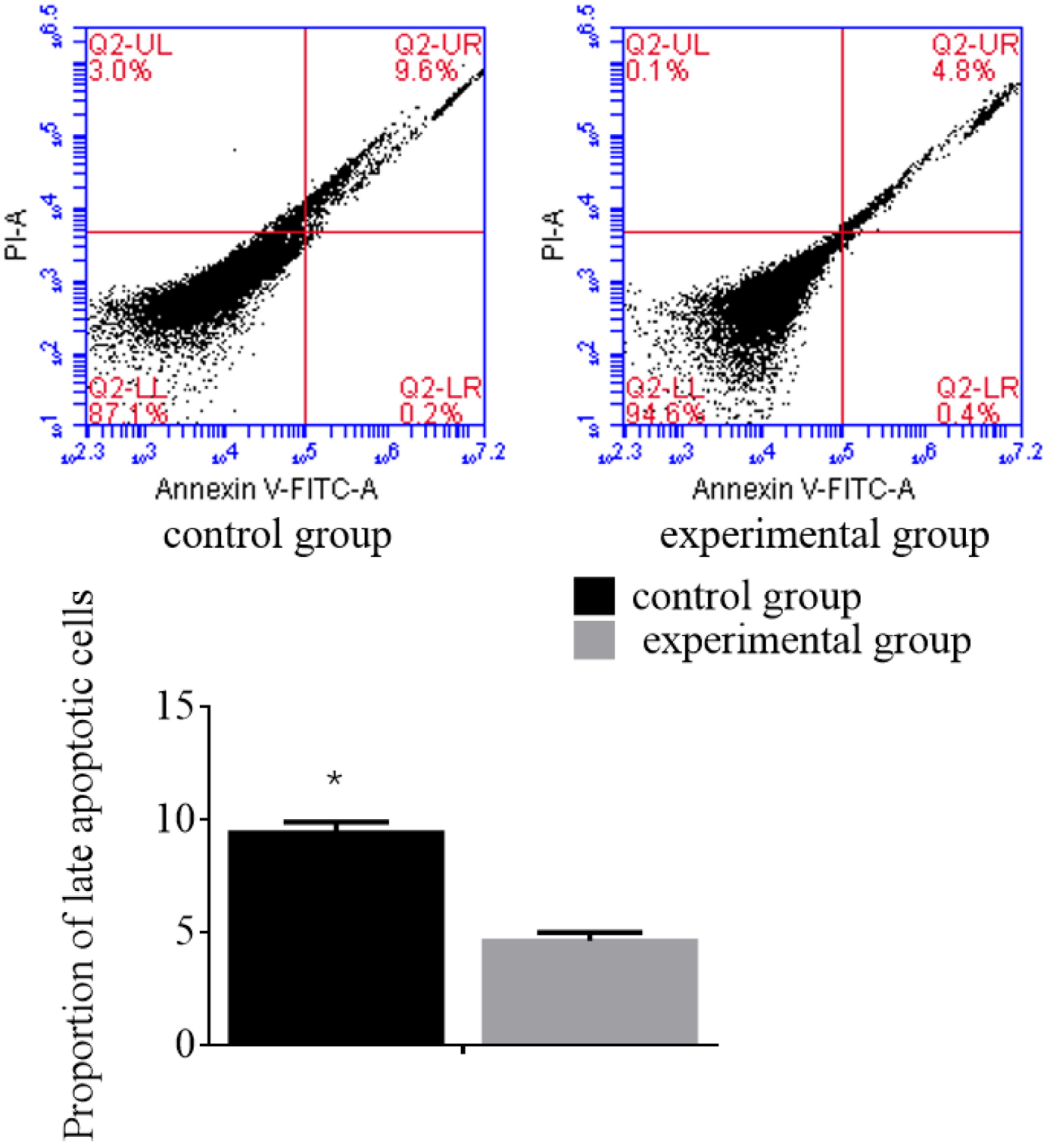
The apoptosis levels in HCC patients from different groups were detected using Flow cytometry assays; *p Compared to the control group, the late apoptotic cell rate in the experimental group was significantly increased and statistically significant (*p* < 0.05).

Flow cytometry was employed to measure the apoptosis levels in HCC cells. The analysis revealed that the experimental group exhibited a significant decrease in apoptosis levels compared to the control group, with a *p* value of < 0.05. These results are presented in Figure 5. The figure displays representative flow cytometry histograms showing the percentage of apoptotic cells in both the experimental and control groups. The experimental group demonstrates a significantly lower percentage of apoptotic cells compared to the control group. These findings indicate that the experimental intervention is associated with a reduction in apoptosis levels in HCC cells, suggesting a potential impact on cell survival mechanisms in these cancer cells.

### 2.16 Effect of E2F1, MDM2, TP53 and CTNNB1 Proteins in HCC

**FIGURE 6:**
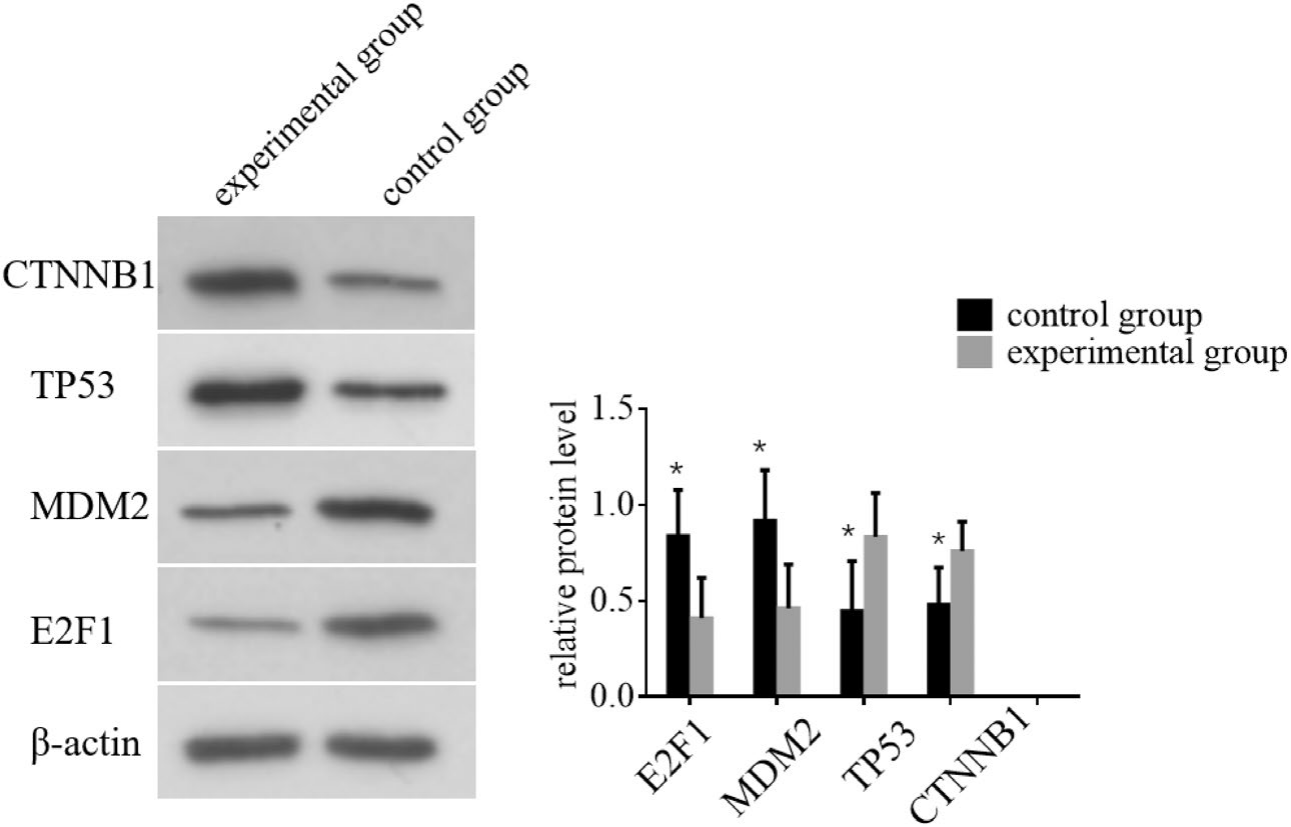
The expression levels of E2F1, MDM2, TP53 and CTNNB1 proteins in HCC patients from different groups were determined using Western blotting. *p indicates that the difference is statistically significant (*p* < 0.05) when compared with the control group.

WB was performed to assess the expression levels of E2F1, MDM2, TP53 and CTNNB1 proteins in HCC patients from different groups. A total of *n* = 10 samples from each group (experimental and control) were analysed. The results indicated significant differences in protein expression. The experimental group exhibited a notable decrease in the expression levels of E2F1 and MDM2 proteins (*p* < 0.05). These findings suggest a downregulation of these proteins in response to the intervention. Conversely, the experimental group showed a significant increase in the expression levels of TP53 and CTNNB1 proteins (*p* < 0.05). This indicates an upregulation of these proteins in the experimental group. These results are summarised in Figure 6. These findings demonstrate that the experimental intervention modulates the expression of key regulatory proteins involved in cell cycle control and tumour progression, highlighting potential mechanisms through which it affects HCC pathology.

### 2.17 Effect of E2F1, MDM2, TP53 and CTNNB1 mRNA in HCC

**FIGURE 7:**
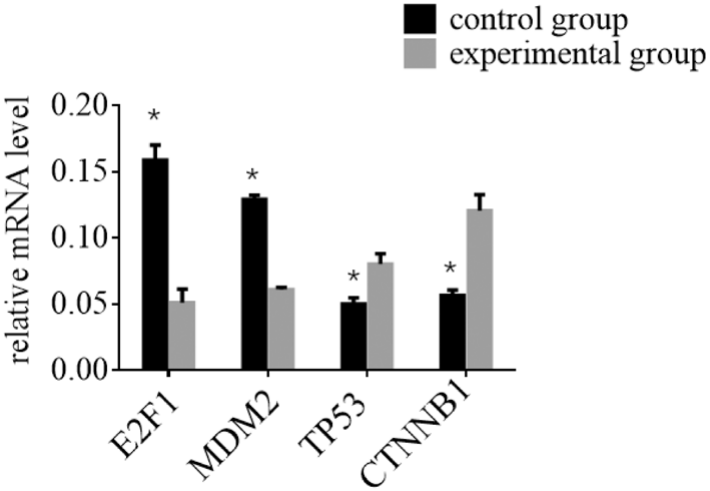
The expression levels of E2F1, MDM2, TP53 and CTNNB1 mRNA in HCC patients from different groups were determined using qRT‐PCR. *p indicates that the difference is statistically significant (*p* < 0.05) when compared to the control group.

To evaluate the expression levels of E2F1, MDM2, TP53 and CTNNB1 mRNA in HCC patients from various groups, we conducted qRT‐PCR. The data revealed significant differences in protein expression between the experimental and control groups. Specifically, the experimental group showed a marked reduction in E2F1 and MDM2 mRNA levels compared to the control group (*p* < 0.05), indicating downregulation of these mRNAs due to the intervention. Conversely, the experimental group exhibited a significant upsurge in TP53 and CTNNB1 mRNA levels relative to the control group (*p* < 0.05), suggesting upregulation of these mRNAs. These findings are illustrated in Figure 7. The results underscore that the experimental treatment influences the expression of crucial regulatory mRNAs associated with cell cycle regulation and tumour progression, shedding light on potential mechanisms by which it impacts HCC pathology.

## 3 Discussion

Our findings provide compelling evidence for the beneficial effects of the experimental intervention on liver tumour pathology in HCC patients. The identification of six key differential metabolites underscores the metabolic shifts associated with improved tumour conditions, which align with emerging research highlighting the role of metabolic alterations in cancer progression and therapy response. Previous studies have noted similar metabolic changes in HCC, suggesting that interventions targeting metabolic pathways could be crucial in enhancing treatment efficacy.

TP53 is a key player in the pathogenesis of HCC, with its mutations leading to the loss of tumour‐suppressive functions and contributing to the progression of liver cancer [16]. The significant upregulation of TP53 in liver tumour tissues observed in our study suggests that the intervention may engage critical tumour suppressor pathways. TP53 is well recognised for its role in cell cycle regulation and apoptosis; its activation can lead to the inhibition of tumour growth and promotion of apoptosis [17]. This finding aligns with literature indicating that restoring TP53 function can be a therapeutic strategy in HCC, where TP53 mutations are prevalent [18]. Our results contribute to this narrative by demonstrating that interventions capable of modulating TP53 expression may offer new avenues for treatment.

The observed inhibition of HCC cell migration further supports the potential of the intervention to reduce invasiveness, a critical characteristic of aggressive tumours. This aspect is particularly relevant, as the metastatic potential of HCC significantly contributes to poor patient outcomes. The reduction in migratory capability we noted may be linked to the modulation of key signalling pathways involved in cell motility, such as those influenced by CTNNB1 (β‐catenin). In prior studies, β‐catenin has been implicated in promoting tumour invasiveness, and the upregulation of CTNNB1 in our experimental group suggests a complex interaction where the intervention may mitigate the pro‐invasive effects of β‐catenin [19, 20].

Reduced levels of key metabolites, particularly those involved in energy production and biosynthetic pathways, can lead to metabolic stress, which has been shown to directly influence the cell cycle. For example, a decrease in ATP or NADPH can activate AMPK (AMP‐activated protein kinase), a cellular energy sensor that induces cell cycle arrest, particularly at the G1/S checkpoint. This cell cycle arrest can act as a protective mechanism to prevent the division of cells under metabolic stress. We believe that this pathway could explain the observed cell cycle alterations in our study. Reduced metabolites, particularly those involved in mitochondrial function (e.g., NAD+, FAD and others), can lead to impaired mitochondrial respiration and oxidative stress, which in turn may activate pro‐apoptotic pathways. Specifically, low energy states or disrupted redox balance can activate Tp53, a critical regulator of apoptosis, as well as other pro‐apoptotic proteins like Bax and Puma. These changes could help explain the increased apoptosis observed in the experimental group, especially if metabolic stress induces a shift towards a pro‐apoptotic cellular phenotype.

Additionally, the differential expression of E2F1 and MDM2 proteins in response to the intervention indicates a shift towards a more favourable tumour microenvironment. E2F1 is known to drive cell proliferation and promote oncogenic processes, while MDM2 acts as a negative regulator of TP53 [21, 22]. The downregulation of these proteins suggests that the intervention not only reduces oncogenic signalling but also enhances tumour suppressive mechanisms. This dual action could explain the observed improvement in the pathological condition of liver tissues.

While our study presents promising insights, it is essential to acknowledge certain limitations. The study design, while robust in demonstrating the intervention's effects, does not fully elucidate the underlying molecular mechanisms at play. Future investigations should delve deeper into the signalling pathways affected by the intervention to clarify these interactions. Moreover, the long‐term implications of the observed metabolic and molecular changes warrant further exploration to assess their relevance in clinical outcomes.

In conclusion, our research contributes to the growing body of evidence supporting the role of targeted interventions in managing HCC. By focusing on the metabolic, apoptotic, and migratory responses of liver tumour cells, we provide a foundation for future studies aimed at optimising therapeutic strategies for HCC patients. The integration of our findings with existing literature highlights the potential for novel treatment paradigms that leverage metabolic modulation and tumour suppressor pathways in the management of liver cancer.

## Author Contributions

**Lai‐wei You:** data curation (equal), formal analysis (equal), investigation (equal), software (equal), supervision (equal), validation (equal), writing – original draft (equal), writing – review and editing (equal). **Jinhuo Wang:** data curation (equal), formal analysis (equal), investigation (equal), methodology (equal), supervision (equal), validation (equal), writing – original draft (equal), writing – review and editing (equal). **Dan Yin:** data curation (equal), formal analysis (equal), methodology (equal), supervision (equal), validation (equal), writing – original draft (equal), writing – review and editing (equal). **Bao‐ji Hu:** data curation (equal), formal analysis (equal), investigation (equal), supervision (equal), validation (equal), writing – review and editing (equal). **Yong Cheng:** formal analysis (equal), investigation (equal), validation (equal), visualization (equal), writing – review and editing (equal). **Xue‐fei Wang:** formal analysis (equal), investigation (equal), supervision (equal), visualization (equal), writing – review and editing (equal). **Hao Li:** data curation (equal), formal analysis (equal), investigation (equal), supervision (equal), validation (equal), writing – review and editing (equal). **Jianrong Guo:** data curation (equal), formal analysis (equal), investigation (equal), supervision (equal), visualization (equal), writing – review and editing (equal).

## Ethics Statement

Ethical approval was given by Gongli Hospital of Shanghai Pudong New Area, and written informed consent was obtained from all patients.

## Consent

All of the authors have consented to publish this research.

## Conflicts of Interest

The authors declare no conflicts of interest.

## Data Availability

The data are freely accessible upon request.
